# Immune functions of pattern recognition receptors in Lepidoptera

**DOI:** 10.3389/fimmu.2023.1203061

**Published:** 2023-06-16

**Authors:** Lin Zhao, Jinlan Niu, Disong Feng, Xialu Wang, Rong Zhang

**Affiliations:** ^1^ School of Life Science and Bio-Pharmaceutics, Shenyang Pharmaceutical University, Shenyang, China; ^2^ School of Medical Devices, Shenyang Pharmaceutical University, Shenyang, China

**Keywords:** pattern recognition receptor, Lepidoptera, pathogen-associated molecular pattern, damage-associated molecular pattern, immune escape

## Abstract

Pattern recognition receptors (PRRs), as the “sensors” in the immune response, play a prominent role in recognizing pathogen-associated molecular patterns (PAMPs) and initiating an effective defense response to pathogens in Lepidoptera. It is becoming increasingly clear that damage-associated molecular patterns (DAMPs) normally play a physiological role within cells; however, when exposed to extracellular, they may become “part-time” critical signals of the immune response. Based on research in recent years, we review herein typical PRRs of Lepidoptera, including peptidoglycan recognition protein (PGRP), gram-negative binding protein (GNBP), β-1,3-glucan recognition protein (βGRP), C-type lectin (CTL), and scavenger receptor (SR). We also outline the ways in which DAMPs participate in the immune response and the correlation between PRRs and immune escape. Taken together, these findings suggest that the role of PRRs in insect innate immunity may be much greater than expected and that it is possible to recognize a broader range of signaling molecules.

## 1 Introduction

Lepidoptera species are the largest phytophagous insects (1), comprising the second largest order of Insecta after Coleoptera (2). Some of the species are economic insects, as represented by *Bombyx mori*, *Antheraea pernyi*, and *Thitarodes xiaojinensis*, whereas others are typical pests in agriculture, such as *Helicoverpa armigera*, *Spodoptera exigua*, *Manduca sexta*, and *Galleria mellonella*. It is noteworthy that there are 472 species of edible insects in sub-Saharan Africa, of which 31% are members of Lepidoptera (3). Meanwhile, Lepidopterans were first applied in the study of innate immunity (4), and emerging data suggest that they also meet the expectations of researchers for using the “3R” principle (replacement, reduction and refinement) in animal experimentation (5). During the long process of evolution, insects have relied on their innate immune system, without adaptive immunity, to resist invasion of exogenous pathogens, and this is the natural advantage of insects as attractive models for studying innate immunity. There are also certain vital reasons, as follows. (i) The virulence factors of human pathogenic microorganisms are similar between insects and mammals, and their virulence is equivalent (6). (ii) Pathogens infect insects and mammals *via* identical mechanisms, including adhesion, invasion, systemic transmission and evasion of the immune response (7). (iii) The physical barrier and innate immune system of insects and mammals show a high degree of homology in function (8, 9). (iv) Insects are easy to breed, convenient to manipulate and economical. For a long time, *Drosophila* has been the chief insect model in gene level research; as a mini-host, it is advantageous for use for forward and reverse genetics (6). However, it also has some shortcomings, such as the inability to be propagated at 37°C, a small size and hemolymph volume (7); in addition, a wealth of operating experience and special laboratory equipment are needed (e.g., microsyringes) (10). Lepidopterans arguably have more advantages in the study of protein levels, with consideration of physiological and immune characteristics. First, it is easier to extract tissues and collect more hemolymph due to the larger size of Lepidopteran larvae. In addition, it is known that the larvae of *G. mellonella* can be propagated at 37°C, which is equivalent to the body temperature of the mammalian host (5). As temperature has been proven to play a significant role in expression of virulence factors, this feature is extremely important in analyzing the innate immune response to pathogens (11).

The innate immune system in insects is composed of two arms: humoral and cellular mechanisms. Humoral immunity entails conversion of prophenoloxidase (PPO) to active phenoloxidase (PO) (12, 13) and expression of genes encoding antimicrobial peptides (AMPs) (14–17) through the Toll and IMD pathways. The above two pathways share many similar features with the Toll-like receptor (TLR) and tumor necrosis factor-α (TNF-α) receptor signaling cascades in mammals (18, 19). Active PO catalyzes conversion of phenols to quinones and promotes formation of melanin, which then participates in cellular immunity (20, 21). AMP directly kills invading microorganisms by interacting with microbial membranes, destroying membrane structures and interfering with internal mechanisms (14, 22). Cellular immunity is mainly mediated *via* hemocytes. If the pathogen is able to breach the physical barriers of the host, hemocytes will be recruited to the site of infection, phagocytosing or killing the pathogens at the membrane or intracellular level (14). In addition, hemocytes and pathogens form microaggregates (21, 23), which accumulate into nodules that are directly encapsulated and eliminated by melanin synthesized by active PO. Pattern recognition receptors (PRRs) are the “sensor” in the immune response that initiates the above two aspects; that is, they are a class of proteins expressed by innate immune cells that recognize invasive pathogens. This recognition involves a process of distinguishing “self” and “nonself” together with the conservative structure of pathogens (bacteria, fungi, viruses and other pathogenic microorganisms have specific structural components completely different from the host body), which are ordinarily referred to as pathogen-related molecular patterns (PAMPs) (24, 25). The role of this recognition process is also the basis of the classical concept of innate immunity (26). In general, PAMPs are mostly located on the cell surface of pathogens, with a few located inside cells. PAMPs can be divided according to their chemical nature into the following categories: (i) polysaccharide compounds, such as peptidoglycan (PGN), β-1,3-glucan, zymosan, lipopolysaccharide (LPS), and capsular polysaccharide (27–29); (ii) lipid compounds, such as LPS, lipoteichoic acid (LTA), and lipoarabinomannan (LAM) (29–31); (iii) proteins and polypeptides, such as flagellin and capsid protein (32); and (iv) nucleic acids, such as dsRNA, ssRNA of viruses and unmethylated CpG of bacteria (33).

Increasing evidence shows that PRRs play crucial roles in identifying endogenous molecules released by damaged cells, which are called damage-associated molecular patterns (DAMPs) (24). A cornerstone concept of DAMPs is that the molecules that act in response to various stresses and damage are host derived rather than pathogen or environment derived (34). DAMPs are regarded as endogenous danger signals because they induce aseptic inflammation without infection and induce an innate immune response that is similar to the response caused by PAMPs (35–37). Although the theory of DAMPs was advanced earlier, in 1994, Polly Matzinger proposed the “danger” theory under the assumption that injured tissues release intracellular molecules to activate the immune system (38). It was not until the high mobility group box 1 (HMGB1) (39), uric acid crystals (40), and Hsp family (41) were successively considered to be DAMPs that this theory began to be widely applied. In the past 30 years, more than 30 types of DAMPs have been confirmed in mammals, located outside the cell (e.g., lmw hyaluronic acid; fibrinogen fibrinogen) or intracellularly, including particles represented by defensins and substances distributed in various structures, such as the cytosol (e.g., S100 proteins, heat shock proteins), nucleus (e.g., HMGB1, DNA, RNA), mitochondria (e.g., mtDNA, formyl peptide), ER (e.g., calreticulin), and plasma membrane (e.g., Syndecans, Glypicans) (37). Dorsal switch protein 1 (DSP1) is a novel kind of DAMP in insects and an ortholog of mammalian HMGB1. Although there are currently few confirmed insect DAMPs, it was recently reported that DSP1 participates in activating the immune response in *S. exigua* after *Bacillus thuringiensis* (Bt) infection (42), providing concrete evidence for the study of insect DAMPs.

PAMPs and DAMPs, either exogenous or endogenous, are danger signals for the body. Detecting them rapidly and responding in time is the key process for insects to initiate the innate immune response and maintain homeostasis. PRRs are the “sensors” that initiate the immunoreaction. Here, we review new findings on pattern recognition receptors in Lepidoptera. In addition, we summarize the research progress of insect DAMPs in recent years. Our purpose is to deepen understanding of the mechanism of innate immunity of insects and reveal possible problems (**Table 1**).

**Table 1:** The number of four types of PRRs involved in Lepidoptera.

Proteins	Lepidoptera	Number	Functions
PGRP	*B. mori*	12	i. Recognition: *Bm*PGRP2-1 (43), *Bm*PGRP-L1 (44) ii. Activate the proPO system: *Bm*PGRP-1 (45), *Tx*PRGP-S2 (46), *Of*PGRP-S (47) iii. Regulate the expression of AMPs: *Px*PGRP-S1 (48), *Of*PGRP6 (49) iv. Amidase activity: *Of*PGRP4 (50), *Ms*PGRP-S1 (51) v. Agglutination: *Px*PGRP-S2 (52) vi. Antiviral: *Se*PGRP-LB (53)
	*T.xiaojinensis*	9	
	*M. sexta*	14	
	*H. armigera*	9	
	*P. xylostella*	9	
	*Ostrinia furnacalis*	14	
βGRP	*B. mori*	4	i. Recognition: *Bm*GNBP (54) ii. Activate the proPO system: *Tx*βGRP1 (55), *Of*βGRP3 (56) iii. Antiviral: *Bm*βGRP4 (57)
	*T. xiaojinensis*	4	
	*M. sexta*	5	
	*H. armigera*	5	
	*P. xylostella*	18	
	*O. furnacalis*	4	
CTL	*B. mori*	24	i Recognition: *Px*CTL5 (58), *Bm*CTL6 (59) ii. Agglutination: *Bm*CTL5 (60), *Ha*CTL7 (61), *Of*CTL6 *(* 62) iii. Activate the proPO system: *Of*IML-10 (63) iv. Regulates the expression of AMPs: *Ha*CTL3 (64) v. Antiviral: *Bm*IML-2 (65)
	*T. xiaojinensis*	32	
	*M. sexta*	34	
	*H. armigera*	26	
	*P. xylostella*	7	
	*O. furnacalis*	14	
Scavenger receptor	*B. mori*	21	i. Recognition: *Bm*SRB8 (66) ii. Regulates the expression of AMPs: *Bm*SR-C (67) iii. Lipid transport:*Bm*SRB3 (68)
	*T. xiaojinensis*	12	
	*M. sexta*	–	
	*H. armigera*	10	
	*P. xylostella*	15	
	*O. furnacalis*	9	

“-” indicates no information from current references.

## 2 The recognition effect of PRRs on PAMPs

**Figure 1:**
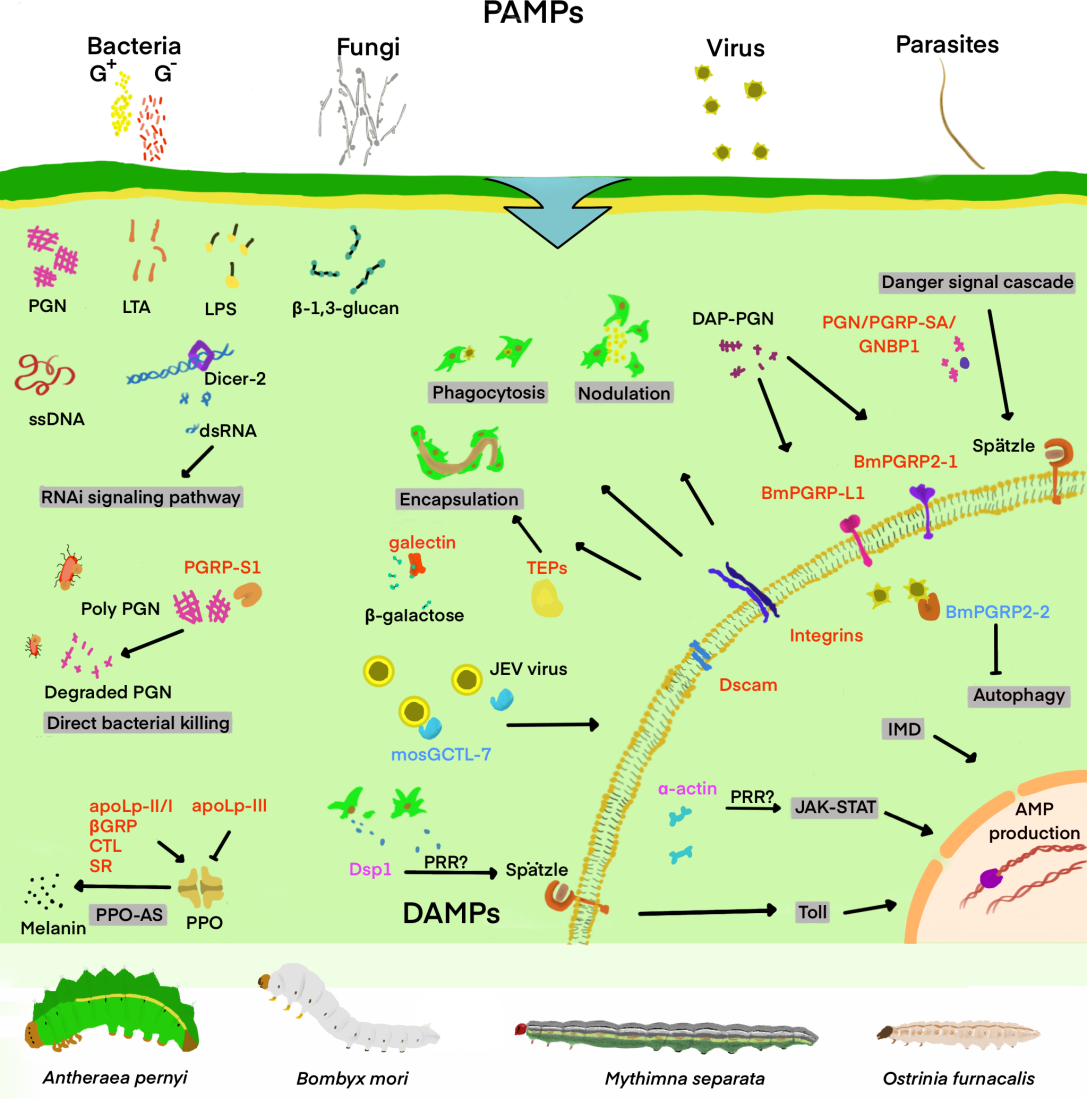
Schematic diagram of important insect’s PRRs involved in the recognition of “danger signal” and the regulation of downstream immune signaling pathways. Infectious invaders include viruses, fungi, gram-positive bacteria (G+), gram-negative bacteria (G-), and other parasites. DAMPs and PAMPs refer to injury-related and pathogen-related molecular patterns, respectively. Some of the PAMPs and DAMPs shown in the figure include double-stranded RNA (dsRNA), single-stranded DNA (ssDNA), β-1,3-glucan, lipopolysaccharide (LPS), lipoteichoic acid (LTA), and peptidoglycan (PGN), Dorsal switch protein 1(Dsp1), and α-actin. The pattern recognition receptors (PRRs) are classified into the secretory and the transmembrane type, and are marked in red in the figure. The secretory PRRs described here consist of short-type peptidoglycan recognition protein (PGRP-Sx); Gram-negative bacteria-binding protein (GNBP); β-1,3-glucan recognition protein (βGRP); C-type lectin (CTL); Scavenger receptor (SR); Apolipoprotein I-III (ApoLp-II/I, III);and thioester-containing protein (TEP), while transmembrane PRRs mainly include Toll, long-type PGRP (PGRP-Lx), integrins and Down syndrome cell adhesion molecule (Dscam). In addition, TEP is also identified as an opsonin of various immune responses, with the PRRs-triggered or regulated immune responses and signaling pathways mentioned in the review as shaded. Damage-associated mode molecule (DAMP) is labeled as magenta, which transmits signals in cells and regulates downstream immune pathways. The molecules labeled with blue are mosquito galactose-specific C-type lectin 7(mosGCTL-7) and *B. mori* peptidoglycan recognition protein 2-2 (*Bm*PGRP2-2), which are involved in the immune escape of the virus, helping to infect the virus and inhibiting the downstream immune response.

What kind of special structure does a pathogen have? Considering this problem may help us to understand why PRRs can quickly identify pathogens and mediate the host’s immune response. A huge portion of PAMPs, mostly polysaccharides, lipids or the proteins or nucleic acids of viruses, are frequently the basic building blocks of pathogens. Such characteristic molecules render PRRs more efficient and accurate in recognizing PAMPs (**Figure 1**). In this section, Lepidopteran insects are taken as an example to briefly describe new PRRs and the characteristics of their involvement in the immune response, for instance, the type of pathogen they recognize and the type of immune response they mediate.

### 2.1 PGRP

Peptidoglycan recognition protein (PGRP), ubiquitous in vertebrates, mollusks, echinoderms and insects, is a pivotal PRR conserved from insects to mammals (54, 69). It plays a role in recognition, bacteriostasis or sterilization, agglutination, and amidase activity and participates in the immune response as a regulator. PGRPs mainly recognize the characteristic bacterial molecule PGN. PGRPs may also recognize LPS and β-1,3-glucan. PGRP can be divided into two types according to the length of the amino acid sequence: a long type (PGRP-L) and a short type (PGRP-S). The long type is an intracellular and transmembrane protein, and the short type is an extracellular protein (70). Furthermore, PGRPs can be divided into catalytic PGRPs and noncatalytic PGRPs according to the catalytic activity of amidase (71). All four mammalian PGRPs, PGLYRP1, PGLYRP2, PGLYRP 3 and PGLYRP4, have the ability to recognize bacterial PGN (72, 73). Among them, PGLYRP1, PGLYRP3 and PGLYRP4 have a direct bactericidal effect on gram-positive and gram-negative bacteria, but with no enzyme activity; PGLYRP2 exhibits amide enzyme activity to hydrolyze bacterial cell wall PGN (74, 75). There are some reports on the function of PGRP in reptiles. C-turtle-PGRP-S in the aquatic reptiles Chinese soft-shelled turtle *Pelodiscus sinensis* has been found to have PGN-binding and antibacterial activities (76). Research on fish PGRP has also gradually increased. Among the currently known 3 kinds of PGRPs in the orange-spotted grouper *Epinephelus coioides*, PGRP-S has direct antibacterial activity against two pathogens, *Vibrio harveyi and Edwardsiella tarda*, and it also significantly induces activation of NF-κB as a regulatory factor (77). PGRP-L1 and PGRP-L2 recognize and bind to PGN, play a role in activating NF-κB luciferase activity, and significantly inhibit growth of *E. tarda* (78). *Aj*PGRP-S in the echinoderm *Apostichopus japonicus* has agglutination ability, strong antibacterial activity against *Vibrio splendidus*, *V. harveyi*, *Vibrio parahaemolyticus*, *Staphylococcus aureus* as well as *Micrococcus luteus*, and high amidase activity in the presence of Zn^2+^ (79). In the mollusk *Crassostrea gigas*, *Cg*PGRPS2 and *Cg*PGRPS4 recognize a variety of microorganisms and PAMPs, playing a pattern recognition role in the innate immune response of this oyster (80).

Compared with other species, the immune function of PGRP in insects has been studied in depth. In addition to the above functions, PGRP, which is an indispensable part of innate immunity, also participates in regulating synthesis of AMPs and activation of the PO system in insects. Insect PGRP mainly plays a recognition role upstream of the Toll versus Imd pathways (71). DAP-PGN in gram-negative bacteria activates the IMD pathway to produce AMPs, whereas Lys-PGN in gram-positive bacteria plays a role in the Toll pathway (81). In *Drosophila melanogaster*, extracellular PGRP-SA and PGRP-SD stimulate the Toll pathway after infection by gram-positive bacteria, and PGRP-SD promote repositioning of peptidoglycan on the cell surface to upregulate the Imd pathway (82–84). Membrane protein PGRP-LC (85–87) versus extracellular PGRP-LE acts upstream of the Imd pathway. Extracellular PGRP-LE participates in the PO pathway (88, 89), and intracellular PGRP-LE is involved in autophagy to defend against pathogens (e.g., *Listeria monocytogenes*) (90). Recent observations have shown that the affinity of PGRP-SC in *Musca domestica* for various intestinal polysaccharides, including LPS and β-1,3-glucan, is beneficial for maintaining intestinal equilibrium (91). Multiple lines of evidence indicate that PGRPs are closely related to regulation of AMPs in Lepidoptera. *Bm*PGRP2-1 and *Bm*PGRP-L1 in *B. mori* recognize DAP-PGN and then trigger the Imd pathway (43, 44). *Px*PGRP-S1 in *Plutella xylostella* and rPGRP6 in *Ostrinia furnacalis* appear to play a prominent role in synthesis of AMPs (48, 49). Amidase activity (71) is also an effective means for involvement of some PGRPs in the immune response, which is beneficial for destroying pathogens and maintaining immune homeostasis in the host. PGRP-S1 is capable of recognizing DAP-PGN and Lys-PGN and exerting amidase activity to degrade PGN, disrupting the bacterial surface (51). PGRP-S5 in *B. mori* is associated with downregulation of the IMD pathway induced by bacteria in the late stage, which is beneficial to prevent overactivation of the immune response, which might have an adverse effect on the host (92).

Recognition between PGRP and PGN also contributes to the PO pathway and plays multiple roles in cellular immunity. PGRP-S1, which was purified from the hemolymph of *B. mori*, was the first PGRP described. After PGN recognition, the PO pathway is activated (54), and PGRP-S4/S5 in *B. mori* has been proven to recognize PGN and trigger stimulation of the PO pathway (93, 94). PGRP-SA in *A. pernyi* has been shown to be a broad-spectrum pattern recognition receptor involved in activation of the PPO system and production of AMPs (95). PGRP-S1 of *Diaphania pyloalis* (*Walker*) recognizes two kinds of PGN and causes strong agglutination of *Escherichia coli*, *M. luteus* or *S. aureus* in the presence of Zn^2+^ (96).

Presently, most research exploring the role of PGRPs in innate immunity focuses on bacteria and fungi, and only a few studies have shown that PGRPs have the ability to respond to virus invasion. *B. mori* bidensovirus (*Bm*BDV) infection significantly increases expression of *Bm*PGRP-LB and *Bm*PGRP-LE in *B. mori* and activates the Imd pathway (97). Induction of *B. mori* cytoplasmic polyhedrosis virus (*Bm*CPV) increases expression of *Bm*PGRPS2. Furthermore, overexpression of *Bm*PGRPS2 activates the Imd pathway, elevates AMPs, and enhances the ability to resist infection by the virus (98). *Se*PGRP-LB in *S. exigua* plays a similar role in *S. exigua* multiple nucleopolyhedrovirus (*Se*MNPV) infection and induces a significant increase in expression of Relish, a key gene in the IMD immune signaling pathway (53).

### 2.2 βGRP/GNBP

The β-1,3-glucan recognition protein family is one of the most characteristic pattern recognition receptor families in invertebrates and mainly includes βGRP and GNBP (99, 100). At present, this family has been identified mostly from insects and crustaceans; it mainly contributes to the innate immune response by participating in activation of the PO system. In crustaceans, this family is also called β-1,3-glucanase-related protein (BGRP) (101). *Pc*BGRP in *Procambarus clarkii* exhibits strong binding to LPS and β-1,3-glucan and enhances PO activation *in vitro* and *in vivo* (102). The first βGRP isolated and purified was from *B. mori*, and it recognizes β-1,3-glucan in the fungal cell wall and initiates activation of the PPO cascade (103). In *O. furnacalis*, after induction by LPS and laminarin (soluble β-1,3-glucan), the mRNA content of *Of*βGRP3 increases, with the LPS-challenged group showing higher levels than the laminarin-challenged group. *Of*βGRP3 also activates the PO pathway after bacterial infection (56). βGRP1 in *T. xiaojinensis* is an essential receptor for activation of PO induced by *Cordyceps militaris.* However, failure of βGRP1 to recognize *Ophiocordyceps sinensis* is the main reason why the host does not undergo melanization after infection. Immunofluorescence detection has revealed a protective layer that prevents βGRP1 from recognizing fungi. βGRPs not only play a recognition role in Lepidopterans to trigger the innate immune response but also act in synergy with other immunity to promote antifungal defense (104). βGRP1 in *T. xiaojinensis* interacts with immulectin-8 (a kind of lectin) to promote encapsulation of pathogens (105). Although the role of βGRPs in viral infection needs further research, βGRPs are considered to have antiviral potential. This view is substantiated by the observation that overexpression of βGRP4 inhibits proliferation of *B. mori* nucleopolyhedrovirus (*Bm*NPV) in *B. mori* ovary N (BmN) cells (57).

GNBPs also may be involved in resisting pathogens. The first member of the GNBP family was isolated and purified from *B. mori*, and it was named because of its strong binding ability to gram-negative bacteria (*E. coli*) but was later classified as a member of the β-1,3-glucan recognition protein family (106), also known as βGRP2 (107). The homologs GNBP1 and GNBP2 found in *D. melanogaster* are involved in defense against gram-positive bacteria (108–111), and GNBP3 correlates with defense against fungal infection (112, 113). GNBP6 in *S. exigua* significantly upregulates PO activity *in vitro* (114). Current research has shown that the immune response of *S. exigua* to fungal infection requires three PRRs (βGRP-1, βGRP-2 and GNBP3) to activate the Toll signaling pathway (115). In addition, *Px*βGBP is a β-1,3-glucan-binding protein identified in *P. xylostella*, which is similar to βGRP and participates in host immunity to fungal infection. Moreover, interference of *dsPxβGBP* sensitizes *P. xylostella* to *Isaria cicadae* infection (116).

### 2.3 CTL

C-type lectins (CTLs), which are widely distributed in both vertebrates and invertebrates, are the most abundant and diverse superfamily of animal lectins (117). CTLs have been studied extensively in mammals. They are often referred to as C-type lectin receptors (CLRs), which play an important role in identifying and resisting pathogens and maintaining the homeostasis of the intestinal flora. For example, several CLRs, including Dectin-1, Dectin-2, Mincle, and Clecsf8, have been shown to be involved in mouse responses to mycobacteria *via* activation of the spleen tyrosine kinase (Syk)/caspase recruitment domain family member 9 (CARD9) signaling pathway (118, 119). Deletion of Dectin-1 and Dectin-2 affect the environment of the bacterial intestinal microbiota of mice and increase susceptibility to colitis (120). Dectin-1 specifically recognizes fungal cell wall β-glucan and then participates in activating the immune response of mice (121). In addition, CLRs in mammals play diverse roles in the development of inflammation and tumors. For example, Mincle in mice is able to inhibit clearance of dead cells and increase production of proinflammatory cytokines; it is involved in the persistent inflammation induced by cell death after acute kidney injury (122). *In vitro* results have shown that downregulation of DC-SIGN significantly inhibits the proliferation, cell cycle progression, migration and invasion of gastric cancer cells (123), suggesting that the expression level of DC-SIGN correlates positively with the occurrence and development of gastric cancer. However, mouse Dectin-2 and Dectin-3 enhances the phagocytic activity of Kupffer cells in the liver and promotes their phagocytosis and clearance of tumor cells, thereby suppressing liver metastasis of tumor cells (124).

Research on CTLs of other vertebrates mainly focuses on their recognition, agglutination or other functions in the process of pathogenic microorganism infection. In poultry, gene expression of cLL in chickens is significantly upregulated after induction by *Avian Pathogenic Escherichia coli* (APEC), suggesting that as a receptor, cLL may play an important role in innate defense against early pulmonary APEC infection in the host (125, 126). In fish, the expression level of *Om*Lec1 in *Onychostoma macrolepis* is significantly increased after induction by *Aeromonas hydrophila*, showing agglutination activity against bacteria such as *E. coli* and *S. aureus in vitro* (127). *Ss*CTL4 in black rockfish recognizes and agglutinates *E. tarda* and *Vibrio anguillarum* and inhibits *E. tarda* infection while promoting invasion of infectious spleen and kidney necrosis virus (ISKNV) through recognition (128). In addition, *Sm*Lec1 in *Scophthalmus maximus* stimulates renal lymphocyte proliferation and enhances the killing effect of macrophages on bacterial pathogens (129).

In invertebrates, CTLs have attracted much attention due to their abilities to recognize and bind sugar ligands, promote agglutination, participate in cellular immunity, mediate synthesis of AMPs and regulate the PO system (130–132). *Hp*-Lec in *Hemifusus pugilinus* displays a broad spectrum of bacterial agglutination activity and agglutination activity against vertebrate blood cells, as well as antifungal activity against *Aspergillus niger* and *Aspergillus flavus* (133). *Cn*lec-1 in *Chlamys nobilis* is upregulated after induction by three immunostimulants, *V. parahaemolyticus*, LPS and PolyI: C, and plays a role in the body’s immune response (134). *Lv*Lec in *Litopenaeus vannamei* is able to enhance *V. harveyi*-induced phagocytosis of blood cells, increase PO activity, and possibly regulate the immune response of blood cells through the cGMP-PKA pathway (135).

The role of some CTLs derived from Lepidopteran insects in innate immunity against exogenous pathogens has also been fully studied and characterized. The bracoviral-derived C-type lectin BLL2 from *S. exigua* is highly sensitive to a variety of bacteria and agglutinates a broad range of bacterial species (136). IML-10 in *O. furnacalis* binds to the surface of hemocytes and promotes their aggregation, thereby enhancing hemocytic encapsulation (63). 20E-HaEcR-HaUSP, a complex formed by the steroid hormone 20-hydroxyecdysone (20E), ecdysone receptor (*Ha*EcR) and ultraspiracle (*Ha*USP), stimulates expression of CTL1 in *H. armigera*. Subsequently, *Ha*CTL1 participates in cellular immunity (encapsulation and phagocytosis) to resist invading pathogens (nematodes and bacteria) (137). In addition, *Ha*CTL3 promotes phagocytosis of hemocytes by bacteria (such as *E. mundtii*), and AMPs regulated by *Ha*CTL3 exert a bactericidal effect. Both of the above strategies may lead to elimination and inhibition of bacteria in the hemolymph of *H. armigera* (64). CTL-14 in *H. armigera* is capable of recognizing fungal surface polysaccharides, forming aggregates with yeast polysaccharides and *Beauveria bassiana* conidia, and interacting with six melanin-related proteins to produce melanin (138). CTL-S6 in *B. mori* binds bacterial PGN strongly but has weak LPS binding ability. In addition, CTL-S6 is involved in encapsulation, activation of the PO pathway and melanogenesis (130). CTL-5 in *B. mori* has been proven to be an important PRR of the JAK/STAT signaling pathway, which mediates nodule melanization during fungal infection (139). Another CTL in *B. mori*, *Bm*CTL10, binds to a variety of PAMPS and plays a role in enhancing encapsulation, nodulation and phagocytosis. It can also bind to blood cells to upregulate synthesis of AMPs. *Bm*CTL10 even downregulates PPO expression as a modulator to protect the body from certain toxic compounds, maintaining immune homeostasis (140). Although little is known about the antiviral effects of CTLs in Lepidoptera, there is increasing evidence of antiviral potential. Expression of *Ha*-lectin in *H. armigera* is upregulated after *H. armigera* nuclear polyhedrosis virus (*Ha*NPV) induction (141). Two bracovirus-associated lectins, *Se*-BLL2 and *Se*-BLL3, have been successively confirmed in recent years to exert antiviral activity during the immune process of *S. exigua* larvae against baculovirus (142) and *Spodoptera frugiperda* larvae against Junonia coenia densovirus (JcDV) (143). IML-2 in *B. mori* inhibits proliferation of *Bm*NPV by promoting apoptosis (65).

### 2.4 Scavenger receptor

Scavenger receptors (SRs) are a class of transmembrane glycoproteins on the cell surface act as PRRs that directly recognize PAMPs as well as DAMPs and participates in the identification, phagocytosis and elimination of pathogens. SRs are generally divided into 12 categories: SR-A~J. Here, we focus on two categories, SR-B and SR-C. SR-B is a kind of PRR that exists in both vertebrates and invertebrates and mediates an immune response (144, 145). It has been reported that scavenger receptor class B1 (SCARB1), which is a typical SR-B, plays a complex biological function. In humans, SCARB1 serves as an important mediator of cholesterol homeostasis, and mutations in SCARB1 are associated with accelerated development of coronary artery disease (146). In mice, SRB1 regulates lymphocyte proliferation and cytokine production (147). SCARB1 in *Serinus canaria* has been shown to be a mediator of carotenoid uptake (148). *Sm*SRB1 in *S. maximus* recognizes *Streptococcus iniae*, *V. anguillarum*, iridovirus, and multiple PAMPs. In addition, it may act as a coreceptor for TLRs and NLRs to regulate immune responses to pathogens (149). *Rp*SR-BI in *Ruditapes Philippinarum* was confirmed to be a PRR with broad-spectrum recognition that may be used as an opsonin to participate in the innate immune response and enhance phagocytosis and chemotaxis of blood cells (150). *Es*SR-B2 in *Eriocheir sinensis* recognizes gram-positive and gram-negative bacteria, thus enhancing phagocytosis and stimulating expression of AMPs (151). *Aj*SR-B in *A. japonicus* binds a variety of PAMPs and exhibits agglutinative activity against gram-positive and gram-negative bacteria (152). SR-B is also of interest in Lepidopteran insects. *Bm*SCRB8 in *B. mori* enhances the bacterial clearance rate and promotes production of AMPs *in vivo* (66). *Mm*SR-B1, an SR-B family member from *Micropilits mediator* (the natural enemy of many Lepidopteran agricultural pests), correlates significantly with synthesis of AMPs and phagocytosis by hemocytes (153). Interestingly, SR-C is only found in invertebrates and not in vertebrates (154). *Mj*SRC in *Marsupenaeus japonicus* plays an important role in the antibacterial immunity of the host by enhancing phagocytosis and expression of AMPs (155). SR-C in *D. melanogaster* is a PRR associated with recognition and phagocytosis of invading bacteria (*E. coli* versus *S. aureus*) (156). SR-C in *Tenebrio molitor* is involved in phagocytosis of microorganisms such as bacteria (*E. coli* versus *S. aureus*) and fungi (*Canidia albicans*) (157). In recent years, the immune function of SR-C in silkworm was reported for the first time. As a pattern recognition receptor against bacteria, SR-C in *B. mori* recognizes and binds diverse types of PAMPs, especially Lys-type PGN, and initiates the immune response. Moreover, SR-C regulates expression of AMPs by activating Toll signaling (67).

### 2.5 Other PRRs

The understanding of innate immunity prompted the search for PRRs. In addition to the above PRRs, a portion of PRRs exhibit pattern recognition characteristics. Galectins are a type of β-galactoside-binding protein that act as a recognition and effector factor in innate immunity by recognizing polysaccharides on the surface of pathogenic microorganisms (158). It has been reported that Galectin-4 plays a recognition role in the fertilized eggs of the silkworm *B. mori* and can induce bacterial agglutination *in vitro* (159). The complement system is involved in mediating elimination of pathogens at an early stage of mammalian infection (160). Thioester (TE)-containing proteins (TEPs) are highly similar to mammalian C3 (161). Macroglobulin complement-related factor (MCR) in *Aedes aegypti* belongs to the TEP family, which functions with SR to regulate expression of AMPs, thus exerting anti-dengue virus (DENV) activity (162). Down syndrome cell adhesion molecule (Dscam) may be involved in various pathways, such as pathogen-specific recognition, phagocytosis, transduction of immune signals, and regulation of AMPs (163). Dscam in *Anopheles gambiae* mediates phagocytosis after bacterial infection (164).

## 3 The potential identification function of DAMPs by PRRs

In general, understanding of DAMPs is constantly being updated with deep investigation. Initially, DAMPs were expected to verify cell death. Subsequently, DAMPs were thought to be secreted or exposed by living cells that experience life-threatening stress. Recently, DAMPs have been found to be crucial to tissue healing after inflammation (165). DAMPs play a role in autoimmune diseases, osteoarthritis, cardiovascular diseases, neurodegenerative diseases and cancer in mammals. Hsp, ATP, HMGB1 and other typical DAMPs provide reference targets for disease diagnosis and treatment (37). Moreover, HMGB1 and ATP have been identified as prominent molecules for promoting regeneration (165).

HMGB1, which is a highly conserved nuclear protein expressed in mammals, normally functions as a DNA chaperone within cells (165, 166). During stress, HMGB1 is released, which can be used as a DAMP to activate the innate immune response. Toll-like receptor (167) and SR (168) in mammals have a recognition role in this process. DSP1, an ortholog of vertebrate HMGB1 (169, 170), is a type of known insect DAMP first discovered in *D. melanogaster* (169, 171). According to current research on insect DAMPs, we speculate that DSP1, as a DAMP triggering the insect signaling pathway, plays a role in amplifying signals during pathogen infection. In *S. exigua*, *Se*DSP1 is released into the circulatory system after infection by bacteria, and the Toll pathway is activated by triggering Spätzle. Then, AMPs and phospholipase A2 (PLA2) are produced. PLA2 catalyzes synthesis of Eicosanoids, which mediate both the cellular and humoral arms in insects. *Xenorhabdus hominickii* inhibits activation of the Toll signaling pathway by DSP1 (including activation of PLA2), which leads to significant immunosuppression in *S. exigua* (171). *Se*DSP1 also regulates production of reactive oxygen species (ROS) through the DSP1/PLA2/Ca^2+^/dual oxidation (Duox) signaling pathway and participates in the intestinal immune response (172). A similar mechanism has been found in mosquitoes (173).

Actin is another typical DAMP in mammals. Filamentous (F-) actin, an important protein involved in cell movement and contraction, is released by dead cells and recognized by an innate immune receptor called DNGR-1 (also known as CLEC9A, a member of the C-type lectin family) (174–176). Recent evidence has shown that α-actin, a cytoskeletal protein closely related to F-actin, is the key trigger for STAT activation in the JAK/STAT pathway in *D. melanogaster* (177).

We speculate that a portion of DAMPs may be conserved between vertebrates and invertebrates, with similar functions and recognized mechanisms. This review provides a basis for follow-up studies on insect DAMPs and shows the possibility of their complementation. Notably, although DAMPs have begun to be discovered in some insects, their paired receptors have not been clarified. What PRRs does DSP1 encounter before triggering Spätzle? In what way is α-actin involved in STAT activation? Are there any proteins in insects that are similar to the PRR that recognizes DAMPs in mammals? These questions are likely to be answered in the future.

## 4 Immune escape

Occasionally, PRRs may act not as an “adversary” but an “accomplice” for pathogens. In mammals, viruses promote invasion by binding to pattern recognition receptors and escape from the innate immune response by interfering with signal transduction and cellular immunity (178). Immune escape by pathogens may lead to persistent infection and even cancer. For instance, Epstein-Barr virus (EBV), which was the first identified human virus associated with tumors, is closely related to development of nasopharyngeal carcinoma (NPC), gastric carcinoma (GC), and several lymphomas. Human papillomavirus (HPV) is associated with cervical, anal, penile, and head and neck squamous cell carcinomas. Furthermore, infection by these viruses is involved in tumor immune escape (47, 179–181). Cancer immune surveillance involves three basic processes of elimination, balance and escape, which is called the theory of cancer immunoediting (182, 183). According to the suggestion of this hypothesis, it is necessary to study the mechanism of immune escape for tumor treatment. Current data indicate that immune escape by pathogens, especially viruses, is closely related to PRRs and that this mechanism exists not only in mammals but also in insects. *Bm*PGRP2-2 in *B. mori*, induced by *Bm*NPV, negatively regulates phosphatase and tensin homolog (PTEN)-phosphoinositide 3-kinase (PI3K)/Akt signaling to inhibit apoptosis to promote replication of the virus (43). As a “bridge”, some CTLs in mosquitoes promote *Flavivirus* infection, such as West Nile virus (WNV) and Japanese encephalitis virus (JEV). The mosGCTL-1/WNV complex formed by mosGCTL-1 in *A. aegypti* and the invading WNV may be captured by the membrane protein mosPTP-1, which is a mosquito homolog of human CD45, at the surface of the cell membrane, promoting invasion by and spread of the virus (184). Studies have confirmed that mosGCTL-7 in mosquitoes is able to bind to the JEV envelope protein *via* an N-glycan at N154 in a calcium-dependent manner and promote viral infection through the mosGCTL-7/mosPTP-1 pathway (185). SRB1 in human hepatocytes and an SRB1-like receptor in mosquito cells acts as cell-binding proteins that bind to and facilitate internalization of DENV and Zika virus (ZIKV) NS1, resulting in high permeability of endothelial cells and downregulation of the innate immune response (186). In addition, knocking out GNBP in *A. aegypti* promotes clearance of DENV-2 (187).

## 5 Discussion

In the long process of evolution, organisms of various taxa have evolved immune systems with multiple structures and functions. Among them, the innate immune system is a common defense mechanism in animals, is especially important in invertebrates, and plays an important role in vertebrates such as zebrafish (188) (**Table 2**). Therefore, as a key link in innate immunity, PRRs have received extensive attention. PRRs of different organisms have both common and surprising characteristics, and they are closely related to each other. Some of the above-described Lepidopteran PRRs are homologs found in higher organisms, and the Toll-like receptors (TLRs) that have been well studied in vertebrates are conserved from *Drosophila* to human (197, 198). Hence, it is important to explore the immune function of PRRs and their association with PRRs of other organisms.

**Table 2:** Comparison between invertebrates and vertebrates.

PRRs	Species	Similarity	Difference
PGRP	Decapoda	–	–
	Lepidoptera	i. Recognition (189) ii. Amidase activity (51, 190) iii. Signal conduction (53, 93, 191, 192) iv. Bacteriostasis or sterilization (74, 193)	Agglutination (96) –
	Primate		–
	Cypriniformes		
βGRP/GNBP	Lepidoptera	i. Recognition function (106) ii. Signal conduction (55, 57)	–
	Decapoda		
CTL	Lepidoptera	i. Recognition (121) ii. Signal conduction (135) iii. Agglutination (127, 194) iv. Promote phagocytosis (137)	–
	Decapoda		
	Primate		
	Cypriniformes		
Scavenger receptor	Lepidoptera	i. Recognition (66) ii. Signal conduction (66)	– mediator of cholesterol homeostasis (146)
	Decapoda		
	Cypriniformes		
	Primate		
TLR	Primate	i. Recognition (195) ii. Signal conduction (196)	–
	Cypriniformes		

“-” indicates no information from current references.

Based on the discussion herein about PRRs of Lepidoptera, the recognition role played by PRRs in insect innate immunity is essential for activation of signaling pathways. Partial PRRs can also directly interact with pathogens, for example, participating in agglutination, cleavage and even elimination. We have noticed that identification of hazardous signals by PRRs is not a single route of transmission but seems to comprise a large closely related network. Furthermore, PRRs form “attack complexes” with other proteins to enhance signal transduction. All of these factors are conducive to the efficient immune response of insects.

As more attention has been given to the role of PRRs in immunity, functional studies are becoming more comprehensive. Some research on PRRs has also been used in breeding of economic insects or formulating pesticides to kill agricultural pests. Here, we illustrate recognition of PRRs for PAMPs and DAMPs but do not discuss their symbiotic relationship or promotion of growth and development. Based on the above, we propose several possible future research directions.

i) Improving understanding of the antiviral pathway of Lepidoptera. Viruses do not have cellular structures, though nucleic acids are currently considered typical of PAMPs, and multiple studies have found that dsRNA, ssRNA, and DNA containing single-stranded unmethylated CpG motifs are recognized by TLR3, TLR7, and TLR9, respectively, in antiviral or antibacterial pathways in mammals (195, 199–202). However, the mechanism of virus recognition in insects is not very clear. For example, *Bm*PGRP-S3 levels are increased after induction by the RNA virus *Bm*CPV, but the specific mechanism needs to be further studied (203). A protein of the viral shell may also play a special role in the recognition process.

ii) Exploring PRRs that bind newly defined PAMPs. In investigations carried out on α-1,3-glucan based on the model of *G. mellonella*, it was validated that *A. niger* α-1,3-glucan, a virulence factor, can play a role in humoral and cellular immunity. Multiple PRRs were also shown to interact with α-1,3-glucan, but Apolipoprotein-III (ApoLp-III) was unable to recognize it. These data provide evidence that α-1,3-glucan is a PAMP that can be recognized by insects and a new direction for enriching understanding of PRRs (204–206).

iii) Searching for Lepidopteran DAMPs and their receptors. The DAMPs reported in Lepidoptera are limited, and PRRs capable of recognizing DAMPs are not clear and need further research.

These issues remind us that continuous attention to risk signaling molecules and pattern recognition receptors capable of recognizing them is indispensable for understanding the innate immunity of the host.

## Author contributions

XW and RZ conceived the concepts, directed the writing and critically revised the manuscript. LZ carried out the manuscript drafting. JN and DF contributed to the design of the tables and figure. All authors have read and approved the content of the manuscript. All authors contributed to the article and approved the submitted version.
